# Gene-editing techniques and their applications in livestock and beyond

**DOI:** 10.5713/ab.22.0383

**Published:** 2023-01-10

**Authors:** Tae Sub Park

**Affiliations:** 1 Graduate School of International Agricultural Technology and Institute of Green-Bio Science and Technology, Seoul National University, Pyeongchang 25354, Korea

**Keywords:** Chicken, CRISPR-Cas9, Economic Traits, Gene-editing, Transgenesis

## Abstract

Genetic modification enables modification of target genes or genome structure in livestock and experimental animals. These technologies have not only advanced bioscience but also improved agricultural productivity. To introduce a foreign transgene, the *piggyBac* transposon element/transposase system could be used for production of transgenic animals and specific target protein-expressing animal cells. In addition, the clustered regularly interspaced short palindromic repeat-CRISPR associated protein 9 (CRISPR-Cas9) system have been utilized to generate chickens with knockout of G0/G1 switch gene 2 (*G0S2*) and myostatin, which are related to lipid deposition and muscle growth, respectively. These experimental chickens could be the invaluable genetic resources to investigate the regulatory pathways and mechanisms of improvement of economic traits such as fat quantity and growth. The gene-edited animals could also be applicable to the livestock industry.

## GENE-EDITING SYSTEMS

### Homologous recombination gene targeting technology

To modify genes or genome structure by knockout of a target gene or its knockin to a locus, gene targeting by homologous recombination is used [1–5]. Homologous recombination enables exchange of genetic information between genomic and exogenous donor templates via crossover events [1]. Combining pluripotent embryonic stem (ES) cells with gene targeting technology has provided insight into gene functions and regulatory mechanisms by enabling the development of knock-out or -in animal models [1–5]. In 1989, mice with knockout of hypoxanthine phosphoribosyltransferase (*HPRT*), *β_2_-microglobulin*, and *c-abl* were generated by germline transmission of knockout ES cells [1–4]. Thereafter, many studies based on homologous recombination for industrial applications and basic research have been published [5]. However, homologous recombination gene targeting is inefficient, costly and involves a time-consuming procedure. In addition, a lack of germline-competent stem cells is a crucial limitation in livestock animals. In 2013, Schusser et al [6] generated immunoglobulin-knockout chickens using chicken primordial germ cells (PGCs) and homologous recombination. Advanced technologies enable site-specific DNA modification using zinc-finger nucleases (ZFNs), transcription activator-like effector nucleases (TALENs), and clustered regularly interspaced short palindromic repeat-CRISPR associated protein 9 (CRISPR-Cas9) [5–8].

### Zinc-finger nuclease and transcription activator-like effector nuclease

Restriction enzymes in bacteria act as genetic scissors that cleave and digest specific DNA sequences. However, restriction enzymes recognize specific sequences of 4 to 8 nucleotides and thus cut at multiple sites in the genome. ZFNs and TALENs allow efficient design and manipulation of a genomic locus compared to homologous recombination [5]. Because ZFNs and TALENs have a conjugated DNA-binding domain and Folk I nuclease domain, they can bind to the DNA sequences and introduce specific genetic alterations or change the genomic structure [5]. Combined with *in vitro* culture and manipulation of chicken PGCs, Park et al [5] produced ovalbumin-knockout chickens using a TALEN. Total proteins in chicken egg white account for about 11% by weight of the egg, and ovalbumin comprises approximately 54% of total egg white proteins [5]. Because of its high protein content, the chicken egg is considered the best system for production of bioactive materials. However, the excessive quantity of ovalbumin hinders the purification of biofunctional proteins. Thus, modification of egg-white components would enable a range of agricultural and industrial applications [5].

### CRISPR/Cas9

The next-generation CRISPR/Cas9 genomic-editing system has revolutionized functional genomics by enabling analysis of gene function *in vivo* and *in vitro* [9,10]. The CRISPR-Cas9 system allows investigation and modification of the biological functions of genes by nucleotide deletion or insertion. In 2013, Zhang and Church groups adapted the bacterial CRISPR-Cas system to genetically modify eukaryotic cells [9,10]. Thereafter, the system was used for gene or genome-editing of bacteria, plants, and animals. Thus, the CRISPR-Cas9 system has been applied to various animal species including experimental animals and chickens, as well as human [9,10].

The CRISPR-Cas9 system is the acquired immune system of bacteria [11]. Upon bacteriophage infection, bacteria dissect and memorize small bacteriophage DNA sequences into the bacterial host genome. Upon re-infection, the bacteriophage is recognized and eliminated by the CRISPR-Cas9 system [11]. The effect is similar to the acquired immunity of eukaryotes [11].

### Production of gene-edited poultry

Genome-edited animals, particularly livestock, will advance agriculture, industry, and bioscience. Generation of genetically engineered livestock for agricultural applications has been reported [12,13]. Whitworth et al [12] generated gene-edited pigs to protect against porcine reproductive and respiratory syndrome virus (PRRSV), which causes huge economic loss globally. Cluster of differentiation 163 (CD163) is the receptor for PRRSV. Whitworth et al [12] produced CD163-knockout pigs using the CRISPR-Cas9 system and examined PRRSV infectivity by virus challenge experiments [12]. CD163-edited pigs showed resistance to PRRSV infection and no clinical signs such as fever or respiratory symptoms [12].

Hornless dairy cattle have been generated for the livestock industry [13]. Physical dehorning of dairy cattle is necessary to protect animals and producers from accidental injury. However, dehorning is costly and raises animal-welfare concerns [13]. Carlson et al [13] identified the hornless locus (known as polled) in polled breeds such as Angus, and replaced the genome structure of Holstein cattle with the polled locus using a TALEN system. Subsequently, polled-edited cattle were produced after nuclear transfer of gene-edited somatic cells [13].

Production of gene-edited mammals could be advanced by combining genetic and cell-engineering techniques such as direct injection into one-cell-stage embryos after *in vitro* fertilization, and stem-cell based or somatic cell nuclear transfer (SCNT)-mediated modification [5,14]. However, application of genetic tools developed in mammals to avian species is hampered by the difficulty involved in accessing the fertilized oocyte nucleus and manipulation of avian oocytes [5,14]. Birds have long been used as model systems in the biological sciences, and so the CRISPR/Cas9 system could advance bioscience and biotechnology research using avian species. Nevertheless, it is technically difficult to generate knockout birds using the CRISPR-Cas9 system because of the developmental and physiological differences between avian and mammalian species [5,14]. Chicken is a major livestock species worldwide; therefore, genome-tailored chickens will not only advance avian biology but also be useful for agricultural and industrial applications (Table 1).

Direct accessibility to embryos during developmental stages has allowed the use of chickens as an animal model for the study of developmental biology and physiology [15]. Since the chicken was the first non-mammal to have its genome sequenced, comparative analysis of the chicken genome has aided in the discovery of novel genes and elucidation of their functions in animals as well as humans [15]. Genome editing technology could advance avian models for various applications. Although knockout chickens generated by homologous recombination have been generated [15], this process is complicated and time-consuming in avian species. Using germline-competent chicken primordial germ cells, which can be maintained *in vitro* without the loss of germ cell properties, it is possible to generate specific gene-tailored chickens without genomic integration of exogenous transgenes [7,8]. Primordial germ cell-mediated germline transmission system has been developed the to generate gene-edited chickens [5,7,8,14]. Primordial germ cells are progenitors of sperms or oocytes after sex differentiation and maturation [5,7,8,14]. Chicken PGCs could be isolated from embryos and expanded them *in vitro*. After co-transfection of cultured chicken PGCs with Cas9 and guide RNA (gRNA) plasmids, the gene-tailored PGCs were transplanted into the blood vessels of recipient embryos. Finally, the gene-edited offspring can be screened and selected by testcross analysis and genotyping [5,7,8,14].

Park et al [7] generated G0/G1 switch gene 2 (*G0S2*)-edited chickens using the CRISPR-Cas9 system. G0S2 is an inhibitor of adipose triglyceride lipase (ATGL, also known as patatin-like phospholipase domain-containing protein 2), which catalyzes the first step of lipolysis (hydrolysis of triacylglycerols into diacylglycerols) [7]. Therefore, it could be hypothesized that the constant reactivity of ATLG enzyme due to elimination of G0S2 inhibition would result in continuous hydrolysis of triacylglycerols in fatty tissue, reducing the lipid content and fat deposition. The excessive body fat of chicken could cause problems with health and production; indeed, fat broiler chickens frequently suffer leg weakness. Other problems such as reduced reproductive performance in the breeding stock can be caused by increased fat deposition. The type of fat in the carcass is not guaranteed in all instances and can lead to health hazards. The next experiment could be designed to investigate the regulatory pathways and mechanisms of metabolism in chicken fat. Additionally, *G0S2*-knockout chickens could be used to investigate obesity as an outbreed animal model for comparison with mouse models.

Kim et al [8] generated myostatin-knockout (*MSTN* KO) chickens using D10A-Cas9 nickase (Cas9n), a mutant Cas9 protein. The D10A-Cas9 nickase system minimizes the off-target effect of a non-specific double-strand break. D10A-Cas9 nickase with the D10A mutation in the RuvC nuclease domain can induce only single-strand cleavage and two gRNAs are needed to induce double-strand cleavage and mutant genotypes [8]. Genetic improvement of economically important traits is a major topic of livestock research. MSTN mutant animals display the double-muscle phenotype with dramatically increased muscle growth [8]. Thus, MSTN is a target for increasing feed efficiency and muscle growth in livestock. Similar to MSTN mutant mammals, MSTN-knockout chickens and quails have significantly larger skeletal muscles [8,16]. Also, muscle hypertrophy and hyperplasia in MSTN-knockout chickens differed depending on sex and muscle type [8,16]. Next-generation sequencing (NGS) of mRNAs from the different muscle types of MSTN-knockout chickens could be applied to investigate differentially expressed genes and regulatory mechanisms. Gene-edited chickens could have applications in a variety of industries. Koslová et al [17] produced avian leukosis virus (ALV)-resistant chickens using the CRIPSR-Cas9 system. ALV-resistant chickens with a premature stop codon were mutated in ALV receptors and were not susceptible to all subgroups of ALV [17].

Gene-edited animals could benefit the livestock industry in the near future. Development of genome-tailored chickens will not only advance avian biology but also go beyond basic research. The candidate gene-targeted platform facilitates precise and efficient genome engineering, and may broaden the range of applications of genome-edited chickens to other industries and as animal models of human diseases.

### Transgenic and gene-edited bioreactor systems

Cell bioreactor systems are designed for the massive production of bioactive materials from animal cell culture and microbial fermentation [18,19]. There are many different types of bioreactors available depending on the desired products and production levels [18,19]. Microbial bioreactors are a relatively rapid and economical way to produce biofunctional proteins compared with animal cell-based systems. It is also easy to scale up microbial fermentation for increased production [18,19]. However, microbial bioreactors are limited in terms of protein conformation, such as the addition of glycosylation features to glycoprotein products and disulfide bond formation after production. This limitation not only affects bioactivity and functionality but can also reduce structural stability *in vivo* [18,19]. Thus, human therapeutic proteins, such as cytokines and antibodies, are mainly produced from animal cell-based bioreactor systems. Animal cell lines such as Chinese hamster ovary (CHO) and human embryonic kidney (HEK293) cells are being used to produce therapeutic proteins with a complete conformation [18,19]. CHO and HEK293 mutant cell lines in which the dihydrofolate reductase (DHFR) or glutamine synthetase (*GS*) gene is mutated for transgene amplification can be used as bioreactors [18,19]. Methotrexate and methionine sulfoximine induce target gene amplification in DHFR and GS knockout cells, respectively [18]. Recently, new gene-edited cell lines were developed using CRISPR-Cas9 technical platforms to improve the efficiency and applicability [18,19]. Mensah et al [19] generated a double knockout of DHFR and DHFR2 in HEK293 cells using the CRISPR/Cas9 system. Since the human genome has two *DHFR* and *DHFR2* genes encoding dihydrofolate reductase activity, the DHFR2 pathway can compensate for compromised DHFR dihydrofolate reductase activity in knockout HEK293 cells [18,19]. As a result, DHFR and DHFR2 double-knockout HEK293 cells showed a significant increase in therapeutic glycoprotein production [18,19]. The CRISPR/Cas9-mediated gene editing system can be used to enhance the proliferative capacity of cell bioreactors through the regulation of cell cycle genes and to improve protein production via the modulation of protein translational circuits.

Animal bioreactors are an alternative system for the production of bioactive proteins, or ‘farmaceuticals’. Transgenic and gene-editing technologies have been employed in an attempt to replicate the high-quality protein deposition systems in the milk of cows and goats or chicken eggs [20]. The expense of animal biopharmaceutical production and the maintenance of bioreactor livestock may be lower than cell culture systems [20]. Advanced technologies such as CRISPR-Cas9 could improve efficiency and productivity in the generation of bioreactor animals. In 2009, ATryn, the brand name of the human anticoagulant antithrombin, was approved by the U.S. Food and Drug Administration (FDA) for the treatment of patients with a hereditary antithrombin deficiency [20]. ATryn is the first drug produced using engineered transgenic animals; genetically modified goats deposit the human anticoagulant protein in their milk [20]. The FDA also approved transgenic chickens genetically engineered to produce sebelipase α (brand name Kanuma) in eggs [20]. As an orphan drug, Kanuma was approved for the treatment of patients with a rare lysosomal acid lipase deficiency in whom the breakdown of fatty molecules is blocked [20].

Herron et al [20] recently demonstrated improved production of human interferon α2a and Fc fusion proteins of macrophage colony-stimulating factor in transgenic chickens. Advanced transgenic animal technologies can improve not only cost-effective production but also the biofunctional reactivity of therapeutic proteins produced in genetically engineered animals. Oishi et al [21] generated bioreactor chickens for human interferon β (hIFN-β) using a CRISPR/Cas9-mediated knock-in system. Oishi et al [21] inserted the *hIFN-β* gene into a specific locus of the chicken ovalbumin gene, encoding a major protein in egg white (approximately 2 g can be found in a single hen’s egg). That report demonstrated the feasibility of a CRISPR/Cas9-mediated knock-in transgenic system at the chicken ovalbumin locus resulting in high-level production of bioactive biomaterials.

The most promising approach for stable integration of a transgene into the host genome is virus-mediated gene transfer [14]. However, viral transduction is restricted for human consumption due to safety issues [14]. Kim et al [22] developed an alternative bioreactor chicken using the non-viral piggyBac transposon and a ubiquitous expression system of human proteins in all body tissues. The transgenic chickens expressed human cystatin-C in all tissues as a result of cytomegalovirus promoter-controlled expression [22]. The large quantity of muscle in chicken could be an alternative candidate for bioreactor target tissue for increased expression and deposition in egg whites. Thus, chicken bioreactors may be a useful and efficient strategy for future applications in agriculture and for biopharmaceuticals with high efficiency and low cost.

### Gene-edited cell line applications

Generated using the CISPR-Cas9 platform, gene- or genome-edited cell lines can be useful for various studies, such as functional genomics. Generally, it is difficult to generate specific gene-targeted livestock due to technical difficulties and the long generation periods of large animals. Thus, gene-edited cell lines from animal tissues can provide versatile approaches to investigate the roles and regulatory mechanisms of specific genes. My lab developed efficient gene-editing systems for chicken genome modulation through a CRISPR-Cas9 system including a D10A mutant Cas9 nickase [7,8,23–25]. The CRISPR-Cas9 system can efficiently and precisely modify genetic information, but non-specific cleavage, known as off-target effects, can induce undesired mutants and frameshifts in other genes can result in biofunctional abnormalities [8,25]. Instead of double-strand DNA breakage for the induction of nonhomologous end joining repair, the mutant nickase creates only a single-strand DNA break, which can reduce non-specific mutant induction without off-target effects [8,25]. Kim et al [8] reported that adaptation of the D10A mutant Cas9 nickase induced 100% mutation efficiency with different mutant genotypes in chicken DF1 cells and caused no off-target effects.

Moreover, for industrial applications, state-of-the-art gene-editing technology could facilitate the development of new fields such as cultured meat production [26]. Cultured meat as a cellular agriculture is a new trend of lab-grown meats produced by *in vitro* cell culture of animal cells [26]. To produce cultured meat, appropriate cell lines and growth media are necessary. Growth media and supplements supply cells with nutrients as well as stimulating and differentiating growth factors, but sustainable growth media for the production of cultured meat is costly and alternative resources for fetal bovine serum, proliferative growth factors, and differentiation-induced components should be developed for industrial applications. Another critical resource is stably proliferative cell lines. Cells can be collected from a primary source of animal tissue via biopsy. However, continuously proliferative cell lines are needed to stably provide cell resources for the production of cultured meat. Transgenic integration of cell cycle regulator genes such as the SV40T large T antigen can produce tumor-like cells with high proliferative ability *in vitro*. These immortalized cells can provide a large quantity of cells for cultured meat production but the use of genome-inserted foreign transgenes for industrial applications is limited due to safety issues. Since Cas9 and gRNA transgenes are generally degraded and disappear after gene modification and the induction of immortalization [7–10], the CRISPR-Cas9 system could be an efficient and safe engineering tool to achieve proliferative and immortal cell lines *in vitro*. Regulatory genes involved in cell proliferation and the cell cycle can be modified and mutated through CRISPR-Cas9 systems to improve proliferation and the muscle tissue-forming capacity for the purpose of cultured meat production.

Additionally, transgenic and gene-edited animal cell lines could be used to improve efficacy and functionality for desirable performance and productivity. miRNAs play critical roles in cell proliferation and differentiation processes by controlling target gene expression [27]. Lee et al [28] reported that the miRNA-mediated knockdown of Forkhead box O3 (FOXO3) in chicken myoblast cells inhibited the cell cycle and proliferation but enhanced myotube differentiation. Interestingly, the knockdown of FOXO3 in chicken myoblasts resulted in no morphological difference at the undifferentiated stage but myotube differentiation was significantly increased in the differentiated condition [28]. Thus, regulation of FOXO3 could be used to improve muscle differentiation for cultured meat production. Lee et al [29] also demonstrated that overexpression of endogenous miRNA-146b-5p in chicken muscle cells modulated cell proliferation and differentiation. Based on mRNA NGS, miRNA-146b-5p could control a transcriptome set related to the cell cycle in chicken muscle cells. Similar to FOXO3, regulation of the miRNA-146b-5p expression pattern could be used to modulate myogenic differentiation *in vitro*. MyoD in particular could be employed to control myogenic activity or proliferative capacity. Kim et al [24] established MyoD knockout quail myoblasts using CRISPR-Cas9. MyoD is a key regulator of muscle differentiation and the dysfunction of MyoD results in repressed myotube formation during myogenesis. MyoD gene-edited mutant quail myoblasts showed no significant difference in paired box protein 7 expression, a marker of undifferentiated myoblasts as well as cell proliferative capacity [24]. However, MyoD knockout cells dramatically suppressed myogenic and myotube formation during differentiation. Overall, specific targeted gene-tailored tools can be efficiently used to modulate cell proliferation and/or differentiation for cultured meat production in the future.

## Figures and Tables

**Table 1:** Summary of the gene-edited chickens and quails

Targeted gene	Knockout or Knockin	Technical platforms	Phenotype	Reference
Immunoglobulin	Knockout	Homologous recombination	No antibody response on immunization	[6]
*OV*	Knockout	TALEN	Chicken ovalbumin gene knockout	[5]
*DDX4*	Knockin	TALEN	Germ cell-specific reporter gene expression	[33]
*G0S2*	Knockout	CRISPR-Cas9	Reduction of fat deposition	[7]
*MSTN*	Knockout	CRISPR-Cas9	Increased muscle growth & reduction of fat deposition	[8] (chicken) [16] (quail)
tva, tvc, tvj	Knockout	CRISPR-Cas9	ALV-resistance	[17]
*OVM*	Knockout	CRISPR-Cas9	No deposition of ovomucoid in egg white	[30]
*DMRT1*	Knockout	CRISPR-Cas9	DMRT1 function as sex determination	[31]
*OV*	Knockin	CRISPR-Cas9	Deposition of hIFN-β in egg white	[21]
Z chr-targeted	Knockin	CRISPR-Cas9	Male-specific GFP expression	[32]

*OV*, ovalbumin; *TALEN*, transcription activator-like effector nucleases; *DDX4*, DEAD-box helicase 4; *G0S2*, G0/G1 switch gene 2; CRISPR-Cas9, clustered regularly interspaced short palindromic repeat-CRISPR associated protein 9; *MSTN*, myostatin; *OVM*, ovomucin; *DMRT1*, doublesex and mab-3 related transcription factor 1; hIFN-β, human interferon β; GFP, green fluorescent protein.
